# Two Decades of *NPM1*-Mutated Acute Myeloid Leukemia: From Molecular Insights to Clinical Decision-Making

**DOI:** 10.3390/cancers18183046

**Published:** 2026-09-19

**Authors:** Gaetano Cimino, Matteo Caridi, Rosanna Celenza, Francesco Millucci, Martina Crocioni, Sofia Sciabolacci, Valeria Cardinali, Maria Paola Martelli

**Affiliations:** 1 Hematology and Clinical Immunology Section, Center for Hemato-Oncology Research, Department of Medicine and Surgery, University of Perugia, 06132 Perugia, Italy; gaetano.cimino@dottorandi.unipg.it (G.C.); rosanna.celenza@uslumbria2.it (R.C.); francesco.millucci@specializzandi.unipg.it (F.M.); martina.crocioni@specializzandi.unipg.it (M.C.); 2 Hematology Division, ‘Santa Maria Della Misericordia’ Hospital, Azienda Ospedaliera di Perugia, 06156 Perugia, Italy; sofia.sciabolacci@ospedale.perugia.it (S.S.); valeria.cardinali@ospedale.perugia.it (V.C.); 3 UOSD Mieloproliferative, Policlinico Tor Vergata, 00133 Rome, Italy; matteo.caridi@uniroma1.it; 4 Department of Physiology and Pharmacology, Sapienza University, 00185 Rome, Italy; 5 Department of Pharmaceutical Services and Clinical Pathology, USL Umbria 2, 05100 Terni, Italy

**Keywords:** acute myeloid leukemia (AML), *nucleophosmin 1* (*NPM1*), molecular biology, targeted therapies, FLT3 inhibitors, menin inhibitors, exportin 1 (XPO1)

## Abstract

*NPM1*-mutated acute myeloid leukemia (AML) represents one of the most frequent and biologically distinct AML entities, characterized by the aberrant cytoplasmic localization of the NPM1 mutant protein. The recent advances in molecular biology and translational research have progressively redefined the clinical management of this disease. This narrative review summarizes the evolution of *NPM1*-mutated AML over the last two decades, focusing on the transition from immunohistochemical observations to modern genetics-based and measurable residual disease (MRD)-driven approaches. We discuss the biological role of mutant NPM1, the prognostic impact of co-occurring genetic alterations, and the integration of molecular monitoring into routine clinical practice. Particular attention is given to the prognostic and therapeutic implications of the co-mutational landscape. We also discuss emerging targeted therapies directed against key pathogenic pathways, including the menin–KMT2A axis and nuclear export machinery. Despite significant advances in risk stratification and treatment, considerable heterogeneity in clinical outcomes persists among patients with *NPM1*-mutated AML, indicating that genomic information obtained at diagnosis alone is insufficient to fully capture prognostic differences. Future advances will likely depend on the integration of genetic, transcriptional, and broader cellular-state information to better define biologically relevant disease states and guide more personalized therapeutic strategies.

## Simple Summary

Acute myeloid leukemia with *NPM1* mutation (*NPM1*-mutated AML) is one of the most frequent and clinically relevant subtypes of acute myeloid leukemia. Over the last two decades, major advances in molecular biology have transformed the understanding of this disease and have directly influenced diagnosis, risk stratification, measurable residual disease monitoring, and treatment strategies. This review summarizes the evolution of *NPM1*-mutated AML from its initial discovery to the present, characterized by precision medicine approaches. We discuss how the identification of co-occurring mutations, the development of molecular monitoring techniques, and the emergence of targeted therapies are reshaping clinical management and improving patient outcomes. We also highlight the limitations of genomic classification alone and discuss emerging approaches aimed at capturing the biological complexity of this AML entity. Indeed, a deeper understanding of disease biology will be essential for developing more personalized and effective therapeutic strategies in the near future.

## 1. Introduction

Acute myeloid leukemia (AML) is a molecularly heterogeneous disease, increasingly classified according to its genetic alterations [1,2]. Among these, *Nucleophosmin 1* (*NPM1*) mutations are the most frequent in adult AML, occurring in approximately 25–35% of de novo cases and in 50–60% of patients with a normal karyotype [3,4,5]. The journey of *NPM1*-mutated AML began with immunohistochemical (IHC) studies in the late 1990s, when the first cases showing aberrant cytoplasmic NPM localization were identified [3,6]. Since then, more than 60 *NPM1* mutations have been described [7]. These mutations result in aberrant cytoplasmic localization of the mutant protein, a biological hallmark of this AML entity [3]. *NPM1* has since become a diagnostic marker, a disease-defining lesion, and one of the most informative molecular markers for MRD monitoring [2,8].

**Figure 1:**
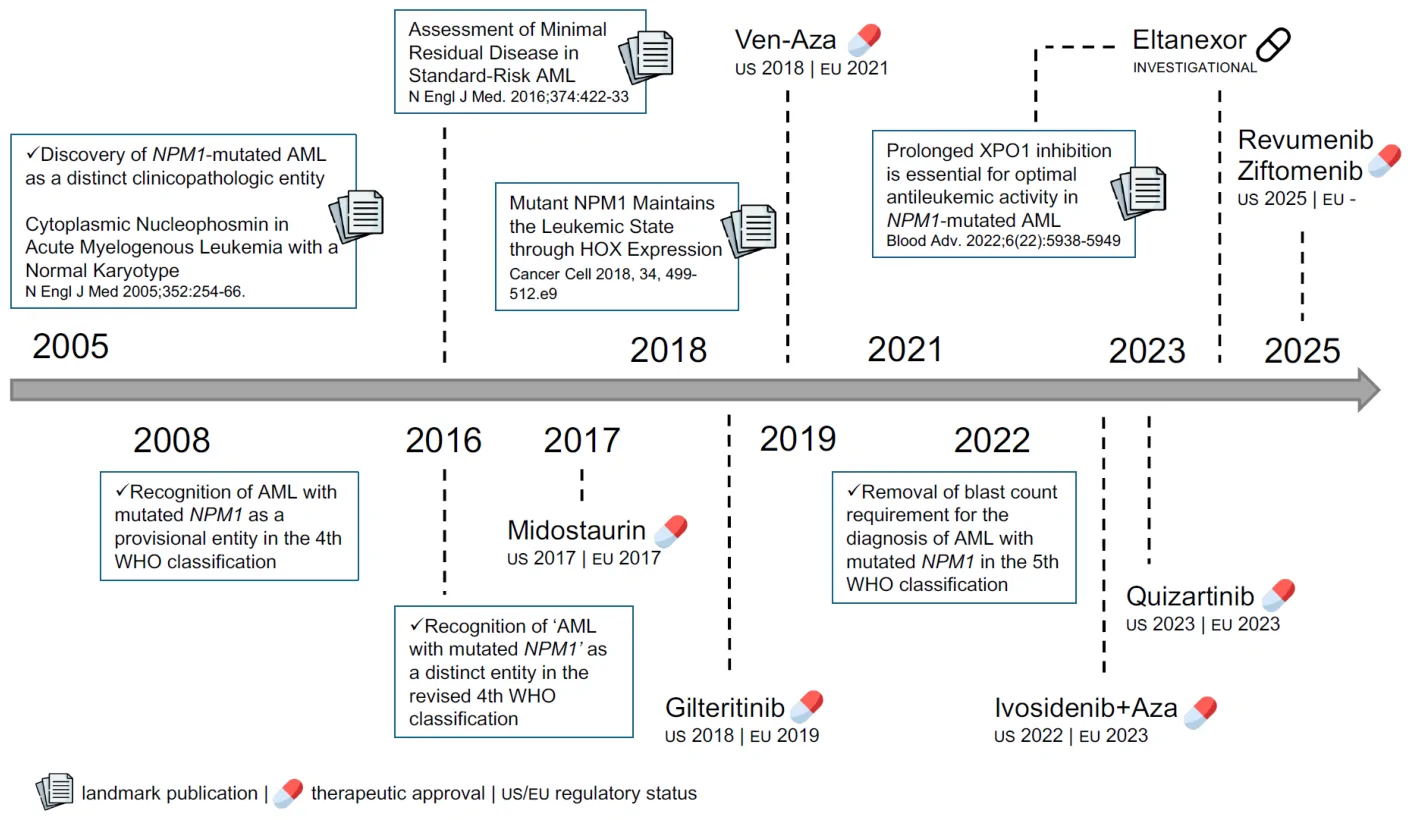
Timeline summarizing the major biological, diagnostic, and therapeutic milestones that shaped the evolution of *NPM1*-mutated AML over the last two decades [3,9,10,11].

Over the past two decades, *NPM1* research has transformed an initial morphological/IHC observation into the recognition of a genetically defined, MRD-trackable, and increasingly therapeutically targetable AML entity (Figure 1). Yet *NPM1*-mutated AML remains biologically and clinically heterogeneous, indicating that baseline genotype alone does not fully explain disease behavior. In this review, we retrace this evolution from the discovery of *NPM1* mutations to contemporary MRD-driven and targeted therapeutic approaches, highlighting the biological and clinical questions that remain unresolved.

## 2. The Discovery of *NPM1* Mutations

NPM1 is a ubiquitous nucleolar phosphoprotein that physiologically shuttles between the nucleus and cytoplasm and acts as a molecular chaperone involved in multiple cellular processes, including ribosome biogenesis, centrosome duplication, genomic stability, and transcriptional regulation [12,13,14].

Before the discovery of *NPM1* mutations in AML, *NPM1* had already been implicated in chromosomal translocations in hematological malignancies [15], including the CD30^+^ anaplastic large-cell lymphoma with t(2;5) [16], the rare MDS/AML with t(3;5) [17], and the exceptional cases of APL with t(5;17) [18,19]. The discovery of *NPM1* gene mutations in AML preceded the era of next-generation sequencing (NGS) and stemmed from the seminal immunohistochemical studies of Falini et al., using monoclonal antibodies against fixative-resistant NPM1 epitopes developed in the hematology laboratories in Perugia [20]. In anaplastic large-cell lymphoma carrying t(2;5), the resulting NPM-ALK fusion protein showed cytoplasmic localization by IHC, suggesting that aberrant cytoplasmic NPM staining could predict underlying *NPM1* genetic alterations [20].

Extending this IHC approach to AML led to the unexpected identification of cases showing aberrant NPM localization in the cytoplasm of leukemic cells. In the seminal study including 591 patients enrolled in the GIMEMA/AML12 EORTC trial, these cases represented a distinctive AML subset (Figure 1) [3]. Notably, most showed a normal karyotype, arguing against a recurrent chromosomal translocation as the mechanism responsible for NPM cytoplasmic localization. These cases also shared characteristic biological features, including frequent myelomonocytic (M4)/monocytic (M5) differentiation, CD34 negativity, and multilineage involvement [3,21]. Subsequent sequencing of the *NPM1* gene, performed in the same laboratories in Perugia, identified recurrent exon 12 mutations, establishing the molecular basis of this originally recognized IHC-defined AML subset [3].

## 3. From IHC to a Genetics-Based Approach

The vast majority (~99%) of *NPM1* mutations are 4-bp insertions within exon 12 [3]. Type A, characterized by a TCTG duplication, accounts for approximately 75–80% of cases, followed by types B and D, whereas other rare exon 12 mutations and rare variants involving exons 5, 9, and 11 have also been described (Table 1) [3,22,23,24,25,26]. Despite their sequence heterogeneity, the characteristic cytoplasmic accumulation of mutant NPM1 results from a dual molecular mechanism [6,27]. *NPM1* mutations typically result in the loss of one or both C-terminal tryptophan residues, W288 and W290, disrupting the nucleolar localization signal (NoLS), together with the acquisition of a novel leucine-rich nuclear export signal (NES). Notably, Nakagawa et al. first pointed out in 2005 that the mutation-generated C-terminal sequence displayed the predicted features of a NES [28]. Subsequent functional studies demonstrated that both acquisition of the NES and disruption of the tryptophan-dependent NoLS are required for aberrant cytoplasmic accumulation of mutant NPM1, with nuclear export mediated by exportin-1 (XPO1/CRM1) [6,27]. However, cytoplasmic accumulation is determined by the balance between the strength of the acquired NES and residual nucleolar retention signals; accordingly, experimental mutants retaining W288 and harboring a weak NES show predominantly nuclear localization [24,29,30]. The acquisition of a functional NES and the consequent interaction of mutant NPM1 with XPO1 represent a key event in NPM1 mutant-driven leukemogenesis [30].

The recurrent structure of exon 12 mutations enabled the development of molecular assays for their detection. The first real-time quantitative polymerase chain reaction (qPCR) assays for *NPM1* mutations were developed in 2006 by Gorello et al. [32]. RNA-based assays specific for type A and type B demonstrated that mutant transcript levels closely paralleled clinical response, providing the basis for the subsequent development of *NPM1*-based molecular MRD monitoring [32]. Together with evidence of multilineage involvement and the relative stability of *NPM1* mutations during disease evolution, these findings established mutant *NPM1* as a robust leukemia-specific molecular marker. This paved the way for molecular detection by DNA fragment analysis and, importantly, for qPCR-based MRD monitoring, now incorporated into ELN recommendations [2,33].

Despite the transition toward molecular diagnostics, IHC retains a valuable complementary role in the diagnostic work-up of patients with AML because of its ability to detect virtually all exon 12 and non-exon 12 *NPM1* mutations and to integrate NPM1 localization with morphological features [34,35].

### 3.1. NPM1-Mutated AML as a Distinct Molecularly Defined Entity

These seminal observations anticipated the subsequent recognition of *NPM1*-mutated AML as a biologically and genetically defined disease entity [36,37,38]. *NPM1*-mutated AML was first introduced as a provisional entity in the 2008 WHO classification [39] and was subsequently established as a distinct entity in the 2016 revision [40]. The 2016 revision further established the primacy of the *NPM1* genotype over morphologic dysplasia, incorporating *NPM1*-mutated cases with multilineage dysplasia into the *NPM1*-mutated AML category [41]. In the WHO 5th edition, the conventional 20% blast threshold has been removed [42], whereas the ICC 2022 retains a threshold of 10% (Figure 1) [1,43]. These changes reflect the biological continuum of *NPM1*-mutated myeloid neoplasia and the high likelihood of progression to overt AML in patients harboring *NPM1* mutation at lower blast counts [44,45,46,47].

### 3.2. MRD-Monitoring in NPM1-Mutated AML

The relative stability of *NPM1* mutations throughout disease evolution makes mutant *NPM1* an ideal leukemia-specific marker for molecular MRD monitoring [9,48,49,50,51,52]. Quantification of mutant *NPM1* transcripts by qPCR, generally expressed as mut*NPM1*/*ABL1* copies (%), represents the reference approach for molecular MRD assessment. According to the ELN-DAVID 2025 recommendations, MRD assessment should include peripheral blood (PB) after two cycles of intensive therapy and bone marrow (BM) at the end of the treatment (EOT) [2,8]. For *NPM1* qPCR, MRD negativity is defined as mut*NPM1*/*ABL1* <0.001% or undetectable. After two cycles, PB levels ≥0.001% and <0.01% are classified as low-level MRD (MRD-LL), whereas levels ≥0.01% are considered MRD-positive. At EOT in BM, MRD-LL encompasses detectable levels ≥0.001% and <0.1%, whereas MRD positivity is defined as ≥0.1%. These thresholds derive from the ELN-DAVID 2025 consensus and should be interpreted in that context. While the ability of MRD-LL to predict relapse remains uncertain, the reduction in EOT BM positivity threshold from the previously recommended 2% to 0.1% allows earlier identification of patients at high risk of treatment failure and potentially broadens the window for pre-emptive intervention [2,8].

Unlike baseline genomic profiling, molecular MRD provides a dynamic measure of treatment sensitivity and residual leukemic burden, thereby refining relapse prediction and post-remission therapeutic decisions. In ELN favorable-risk *NPM1*-mutated/*FLT3*-ITD-negative AML, allo-HSCT in first complete remission is generally not routinely indicated in patients achieving an optimal molecular response. Conversely, persistent MRD may identify patients at higher risk who could benefit from treatment intensification or transplantation. In *NPM1*-mutated AML with concomitant *FLT3*-ITD, currently classified as intermediate risk, post-treatment *NPM1* MRD provides additional information that may help refine the indication for allo-HSCT [53,54]. Whether combined *NPM1* and *FLT3*-ITD MRD negativity can identify patients in whom allo-HSCT may safely be omitted in CR1 remains an important question for prospective investigation.

The prognostic value of molecular MRD also extends to lower-intensity approaches. In a real-world cohort of 76 patients with *NPM1*-mutated AML treated with venetoclax-based combinations, achievement of BM MRD negativity by the end of cycle 4 was associated with a 2-year OS of 84%, compared with 46% in MRD-positive patients, and emerged as the strongest prognostic factor in multivariable analysis [55].

Although molecular MRD is a powerful prognostic marker, whether MRD-guided therapeutic intervention improves survival remains less clearly established. In the UK NCRI AML17/AML19 randomized trials, sequential molecular MRD monitoring coupled with clinician-directed intervention did not improve OS in the overall study population, whereas a survival benefit was observed in patients with *NPM1*-mutated/*FLT3*-ITD AML [56]. These findings do not diminish the established role of MRD in risk stratification, but highlight that the optimal therapeutic intervention triggered by an MRD result remains to be defined. Further prospective randomized studies are warranted to identify the patient subsets most likely to benefit from MRD-guided treatment and to determine which MRD-directed interventions translate into improved survival.

### 3.3. MRD Relapse and Pre-Emptive Therapy

According to the ELN-DAVID 2025 consensus, molecular MRD relapse in *NPM1*-mutated AML is defined as conversion from undetectable to detectable mut*NPM1* with levels ≥0.1% in BM or ≥0.01% in PB. In patients who have never achieved MRD negativity, a ≥1 log10 increase from the previously measured nadir within the same tissue compartment is required [8]. Suspected MRD relapse should be promptly confirmed in a second consecutive sample, preferably using paired PB and BM, within 4 weeks and ideally within 2 weeks. However, in patients at high risk of imminent hematologic relapse, confirmatory testing may not permit sufficient time for intervention, particularly in the presence of BM mut*NPM1*/*ABL1* ≥1%, *FLT3*-ITD MRD ≥0.01% by ultrahigh-sensitivity next-generation sequencing (UHS-NGS), or multiparameter flow cytometry (MFC)-MRD ≥0.1% [8].

Because *NPM1* molecular relapse strongly predicts impending hematologic relapse, increasing evidence supports pre-emptive anti-leukemic treatment at the time of MRD relapse, before overt morphologic recurrence [57,58]. Venetoclax-based regimens have shown encouraging activity in this setting and may provide an effective bridge to allo-HSCT. A similar treatment-intensification strategy may be considered in patients with persistent MRD at EOT, particularly in view of the adverse prognostic impact of peri-transplant MRD positivity; however, management should be individualized according to baseline risk, MRD kinetics, and transplant eligibility [59,60].

### 3.4. Alternative Molecular Approaches for MRD Monitoring

In clinical practice, qPCR-based MRD monitoring is largely restricted to the most common *NPM1* mutation types, particularly A, B, and D, for which standardized plasmid-based calibration curves are available [61]. Consequently, patients harboring rare *NPM1* variants may not be amenable to conventional qPCR-based monitoring.

Digital PCR (dPCR) represents an attractive alternative in this setting because it enables absolute quantification without the need for external standards or plasmid-based calibration curves. Lesieur et al. demonstrated that dPCR can reliably quantify rare *NPM1* mutations, with results comparable to standard qPCR [61]. Although the ELN-DAVID 2025 recommendations provide methodological guidance and dPCR-specific thresholds, the prognostic significance of these cut-offs requires further prospective validation [8].

Additional molecular approaches may provide complementary information in *NPM1*-mutated AML with concomitant *FLT3*-ITD. Although *NPM1* remains the benchmark molecular marker because of its greater biological stability, highly sensitive *FLT3*-ITD MRD assessment can provide additional prognostic information at selected time points, particularly after induction and before allo-HSCT [62,63,64,65]. Discordant *NPM1* and *FLT3*-ITD MRD results may occur, reflecting differences in clonal architecture, disease kinetics, and assay sensitivity; when both markers are available, double MRD negativity generally identifies patients with the most favorable outcome [63]. However, the incremental prognostic value of *FLT3*-ITD over *NPM1* MRD may vary according to the clinical time point and treatment context [65,66]. Thus, in *NPM1*/*FLT3*-ITD co-mutated AML, *FLT3*-ITD MRD should currently be considered complementary to, rather than a replacement for, *NPM1*-based monitoring. A comprehensive discussion of *FLT3*-ITD MRD methodology and its integration with FLT3 inhibitor-based therapies is provided elsewhere in this issue [67].

## 4. Molecular and Immunophenotypic Landscape of *NPM1*-Mutated AML: Prognostic and Therapeutic Implications

Since the seminal description of *NPM1*-mutated AML, this entity has been associated with high response rates to induction chemotherapy and relatively favorable outcomes compared to *NPM1*-wild type AML [3]. However, subsequent studies demonstrated that this favorable effect is strongly influenced by the co-mutational landscape, particularly by the presence of *FLT3*-ITD [31,68,69]. These observations established *NPM1*-mutated AML as a paradigmatic example of how gene–gene interactions can modify prognosis and, increasingly, therapeutic selection.

From a therapeutic perspective, these vulnerabilities can be conceptualized across complementary biological levels. Recurrent co-mutations may create actionable oncogenic dependencies, most notably aberrant FLT3 signaling and the metabolic and epigenetic reprogramming driven by mutant IDH. In parallel, the NPM1 mutant itself establishes core dependencies required for disease maintenance, particularly on the menin–KMT2A–HOX/MEIS1 transcriptional axis and XPO1-mediated nuclear export. Finally, the recurrent expression of surface antigens provides additional opportunities for targeted therapies.

### 4.1. FLT3-ITD and the NPM1/DNMT3A/FLT3-ITD Triple-Mutated Genotype

Among the co-occurring genetic lesions in *NPM1*-mutated AML, *FLT3*-ITD is particularly relevant because of its high frequency, adverse clinical impact, and biological cooperation with mutant NPM1. *FLT3*-ITD is detected in approximately 40% of *NPM1*-mutated AML and has historically been associated with a higher risk of relapse and inferior survival [31,68,69]. *FLT3*-ITD results in constitutive activation of FLT3 signaling, promoting proliferation and survival of hematopoietic progenitors. Experimental models provided compelling evidence for biological cooperation between the two lesions. Vassiliou et al. first demonstrated the leukemogenic potential of *Npm1c* and its cooperation with additional genetic events [70]. Subsequently, two independent murine models directly demonstrated a striking synergy between *Npm1c* and *Flt3*-ITD [71,72]. In the model reported by Mupo et al., compound *Npm1c*/*Flt3*-ITD mice developed rapidly penetrant AML, with all double-mutant animals developing leukemia within 31–68 days, providing a biological rationale for the frequent co-occurrence of these two mutations in human AML.

The prognostic impact of this interaction is further influenced by additional co-mutations. Papaemmanuil et al. identified the co-occurrence of *DNMT3A*, *NPM1*, and *FLT3*-ITD mutations as a high-risk genomic subgroup, associated with an increased risk of death (HR 1.5; 95% CI, 1.2–1.9) [5]. This ‘triple-mutated’ genotype represents the most frequent three-gene co-occurrence in AML, occurring in approximately 6% of patients, and provides a paradigm of gene–gene interaction in which the adverse prognostic impact of *FLT3*-ITD is particularly pronounced compared with its presence in other genomic contexts [5]. Long-term outcomes were markedly inferior, with a 5-year OS of approximately 20–30%, compared with around 50% in patients harboring *NPM1* and *DNMT3A* mutations without *FLT3*-ITD [5].

The incorporation of FLT3 inhibitors into intensive chemotherapy has progressively modified this adverse prognostic landscape. Midostaurin improved outcomes when added to intensive chemotherapy in newly diagnosed *FLT3*-mutated AML, while quizartinib more recently demonstrated a survival benefit specifically in newly diagnosed *FLT3*-ITD AML [73,74]. Recent retrospective subgroup analyses suggest that incorporating FLT3 inhibitors may mitigate the historically adverse prognostic impact associated with the *NPM1*/*DNMT3A*/*FLT3*-ITD triple-mutant genotype; however, prospective studies are needed to validate this observation. [75,76]. Accordingly, the prognostic significance of *FLT3*-ITD should now be interpreted in the context of contemporary FLT3-targeted therapy. In the ELN 2022 classification, *NPM1*-mutated AML with concomitant *FLT3*-ITD is categorized as intermediate risk, irrespective of the *FLT3*-ITD allelic ratio [2].

Importantly, however, *FLT3*-ITD status alone may not be sufficient to determine the indication for allo-HSCT in first complete remission. Post-induction molecular response provides additional clinically relevant risk stratification. In the AML17/AML19 studies, CR1 allo-HSCT was associated with a survival benefit in *NPM1*-mutated patients with persistent molecular MRD, whereas no benefit was observed in patients achieving MRD negativity. Importantly, this finding was maintained among patients with concomitant *FLT3*-ITD: 3-year OS was 45% versus 18% in MRD-positive patients undergoing versus not undergoing CR1 allo-HSCT, whereas no significant benefit was observed in MRD-negative patients (83% versus 76%) [77]. These findings support the integration of baseline *FLT3*-ITD status with post-treatment *NPM1* MRD when defining post-remission strategies. Emerging data further suggest that highly sensitive *FLT3*-ITD MRD may help identify patients more likely to benefit from post-transplant FLT3-inhibitor maintenance, although prospective validation of MRD-guided treatment selection remains required [78,79].

**Figure 2:**
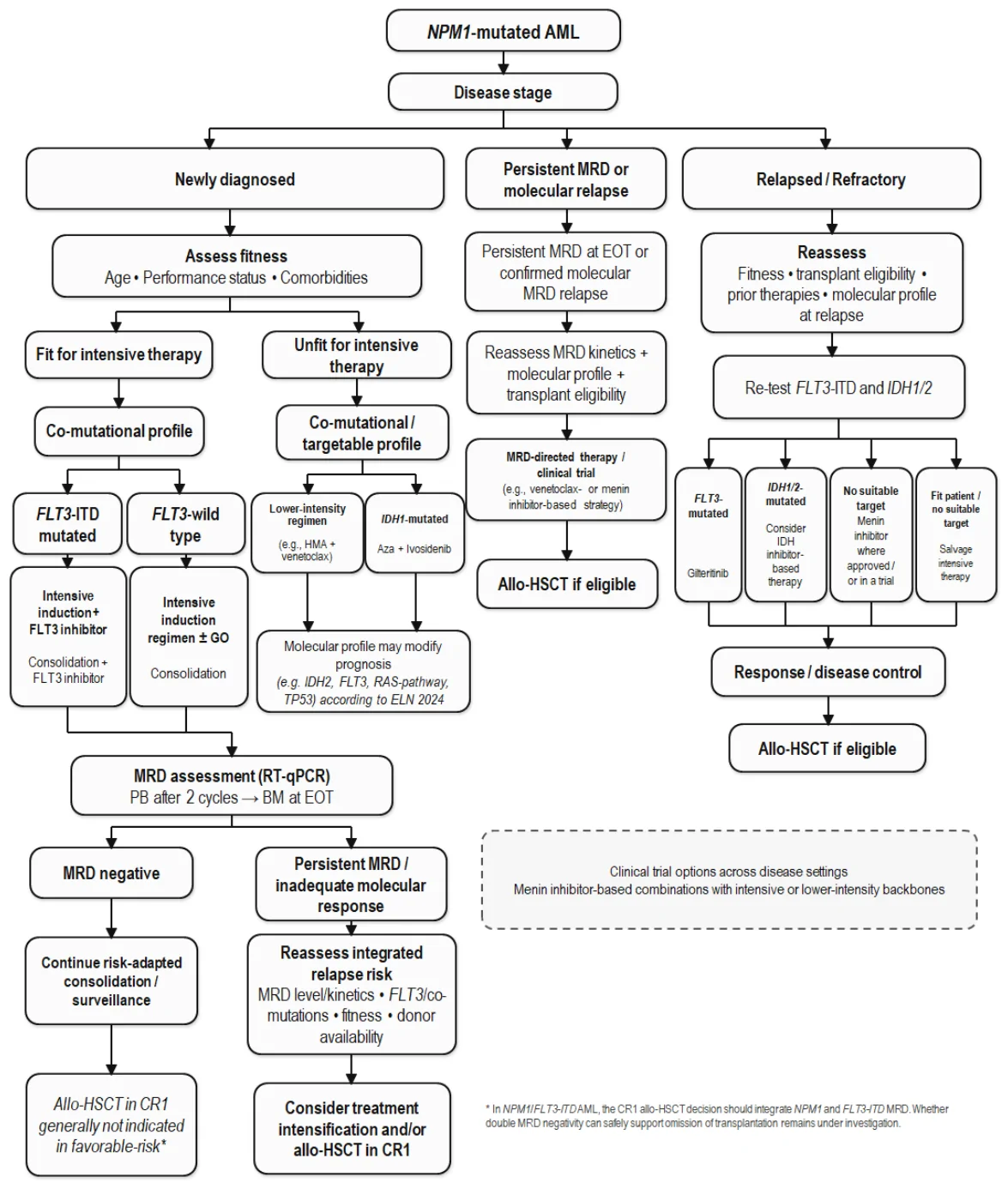
Illustrative therapeutic framework for *NPM1*-mutated AML according to disease setting, patient fitness, molecular profile, and MRD response. Treatment options are non-exhaustive and should be individualized according to current regulatory approvals, guideline recommendations, prior therapy, transplant eligibility, and clinical trial availability. Clinical trial enrollment, including studies evaluating menin inhibitor-based combinations with intensive or lower-intensity backbones, may be considered across disease settings.

In relapsed/refractory *FLT3*-mutated AML, the introduction of gilteritinib represented a further major therapeutic advance. In the ADMIRAL trial, median OS was 9.3 months with gilteritinib compared with 5.6 months with salvage chemotherapy (HR 0.64; *p* < 0.001), with composite complete remission rates of 34.0% and 15.3%, respectively [80]. Notably, molecular profiling of the ADMIRAL population showed that patients harboring concomitant *NPM1* and *DNMT3A* mutations who received gilteritinib had the most favorable outcomes among the molecular subgroups analyzed [81]. This observation further supports FLT3 inhibition as a particularly relevant therapeutic strategy in the molecular context of *NPM1*/*DNMT3A*/*FLT3*-mutated AML (Figure 2).

Finally, the conventional binary distinction between *FLT3*-ITD-positive and -negative disease may itself be challenged by increasingly sensitive molecular approaches. Recent data indicate that low-level *FLT3*-ITD microclones below conventional diagnostic thresholds are enriched in *NPM1*-mutated AML and are associated with an increased risk of relapse [82]. Thus, a subset of patients conventionally classified as *FLT3*-ITD-negative may still harbor biologically and clinically relevant *FLT3*-ITD subclones.

### 4.2. Beyond FLT3-ITD: Heterogeneity Within Favorable-Risk NPM1-Mutated AML

According to the ELN 2022 classification, *NPM1*-mutated AML without *FLT3*-ITD is generally assigned to the favorable-risk category, in the absence of adverse-risk cytogenetic abnormalities [2]. However, this apparently homogeneous group remains clinically heterogeneous, with a subset of patients experiencing treatment resistance or relapse despite favorable baseline genetic classification. Within this genetically heterogeneous group, some co-mutations primarily modify prognosis, whereas others may create biologically distinct and actionable oncogenic dependencies. Identifying the biological determinants of this residual high-risk population therefore remains an important unmet need. The ELN itself recognized that the prognostic impact of additional molecular abnormalities, including myelodysplasia-related gene mutations, within *NPM1*-mutated AML remained to be fully defined [2].

Early genomic studies already indicated that the prognostic impact of *NPM1* mutations depends on the broader co-mutational context. Patel et al. showed in 2012 that *IDH1/2* mutations significantly co-occur with *NPM1* and that specific combinations were associated with markedly different outcomes [83]. In the *FLT3*-ITD-negative intermediate-risk population analyzed in that study, the 3-year OS was 89% in patients harboring both *NPM1* and *IDH1/2* mutations compared with 31% in *NPM1*-mutated/*IDH1/2*-wild-type patients [83]. More recent genomic profiling confirms the close association between these lesions: in the AMLSG 09-09 cohort, *IDH1* and *IDH2* mutations were detected in 18% and 21.3% of *NPM1*-mutated AML, respectively [84].

This association also has a biological basis. Experimental studies have demonstrated functional cooperation between mutant NPM1 and IDH. In a murine AML model, *IDH2*R140Q cooperated with NPM1c through activation of HOXA9/MEIS1- and hypoxia-related programs, while genetic suppression of mutant *IDH2* impaired leukemia stem-cell maintenance [85]. More recently, a double-knock-in model showed that *Idh1*R132H combined with *Npm1c* was sufficient to induce overt AML, further supporting a genotype-specific leukemogenic cooperation between these lesions [86].

Beyond their biological cooperation, *IDH1/2* mutations generate therapeutically exploitable metabolic dependencies. Mutant IDH enzymes produce the oncometabolite (R)-2-hydroxyglutarate (2-HG), which alters mitochondrial metabolism and contributes to dependence on the anti-apoptotic protein BCL-2 [87]. This vulnerability is consistent with the favorable outcomes observed in patients with *IDH1/2*-mutated AML treated with venetoclax plus azacitidine (Ven/Aza) [88,89], including selected younger patients in whom this combination has been used as a bridge to allo-HSCT [90].

*IDH* mutations also provide an opportunity for direct molecular targeting. In patients with newly diagnosed *IDH1*-mutated AML who are ineligible for intensive chemotherapy, ivosidenib plus azacitidine significantly improved event-free survival compared with placebo plus azacitidine in the AGILE trial [91], with a median OS of 29.3 months in subsequent follow-up [92]. Ivosidenib plus azacitidine therefore represents a frontline therapeutic option in this setting (Figure 2) [2,93]. In relapsed/refractory *IDH2*-mutated AML, enasidenib achieved a CR rate of approximately 19% and a median OS of 9.3 months [94], although it is not currently approved in Europe.

Other non-*FLT3* co-mutations have raised important questions regarding risk stratification rather than direct therapeutic targeting. Secondary-type mutations, now commonly referred to as myelodysplasia-related (MR) gene mutations, include alterations involving *SRSF2*, *SF3B1*, *U2AF1*, *ZRSR2*, *ASXL1*, *EZH2*, *BCOR*, and *STAG2*. In their seminal 2015 study, Lindsley et al. showed that these lesions were strongly associated with secondary AML and frequently represented early events acquired during preleukemic evolution [95]. Their prognostic significance in *NPM1*-mutated AML, however, has remained controversial, with different studies reporting discordant effects [96].

This issue was recently addressed by Cocciardi et al. in 568 intensively treated patients with *NPM1*-mutated AML enrolled in the AMLSG 09-09 trial [84]. MR gene mutations were identified in approximately 18% of patients and were particularly enriched in older individuals. Among the 470 patients classified as ELN 2022 favorable risk, MR gene mutations were associated with inferior EFS in multivariable analysis. Importantly, however, when post-cycle 2 *NPM1* MRD status was incorporated into landmark analyses, the adverse prognostic effect of MR gene mutations was no longer significant, whereas molecular response retained strong prognostic relevance [84].

Beyond the grouped analysis of MR gene mutations, the prognostic impact of individual co-mutations was heterogeneous: *DNMT3A* R882 and *MYC* retained adverse prognostic effects, whereas cohesin gene mutations remained associated with favorable outcome after adjustment for MRD [84].

These findings indicate that post-treatment *NPM1* MRD can substantially refine, and in some settings supersede, prognostic associations derived from baseline mutational profiling. However, MRD identifies which patients retain residual disease rather than fully explaining the biological mechanisms underlying treatment resistance.

Emerging transcriptomic and multiomic studies are beginning to address this question. Single-cell and transcriptomic profiling has identified biologically distinct classes within *NPM1*-mutated AML characterized by different cellular states, immune-evasion programs, and markedly different post-transplant outcomes [97,98]. More recently, integrated analysis across 13 genomic, transcriptomic, proteomic, post-translational, metabolic and lipidomic modalities identified three distinct *NPM1*-mutated AML subtypes, differing in cellular differentiation state, co-mutational architecture and potential therapeutic vulnerabilities [99]. These emerging data suggest that the next step in risk stratification may require integration of baseline genetics, dynamic MRD response and broader transcriptional, cellular and microenvironmental states, although their clinical utility requires prospective validation.

### 4.3. CD33 and CD123 as Therapeutic Targets in NPM1-Mutated AML

Beyond its distinctive genetic architecture, *NPM1*-mutated AML is characterized by recurrent immunophenotypic features with relevant therapeutic implications [100]. Among these features, high CD33 expression is frequently observed in leukemic cells carrying *NPM1* mutations, providing a strong biological rationale for the use of gemtuzumab ozogamicin (GO), an anti-CD33 antibody–drug conjugate, in this AML entity [101]. The clinical activity of GO has been supported by randomized studies in newly diagnosed AML, including ALFA-0701 [102,103], and, more specifically, by the *NPM1*-mutated AMLSG 09-09 phase III trial [104]. Although the latter did not demonstrate a significant improvement in its primary EFS and OS endpoints, GO significantly reduced the cumulative incidence of relapse. Moreover, molecular analyses showed deeper *NPM1* MRD responses, with higher rates of MRD negativity at EOT and a lower relapse incidence, further supporting the biological relevance of CD33 targeting in *NPM1*-mutated AML [104].

CD123, the α chain of the interleukin-3 receptor, represents another attractive therapeutic target in this disease. Perriello et al. showed that CD123 is consistently highly expressed on *NPM1*-mutated AML cells [105], including the CD34+CD38- leukemic compartment, a phenotypically immature fraction previously shown to contain *NPM1*-mutated leukemia-initiating cells [106], with CD123 expression further increased in cases harboring concomitant *FLT3* mutations [105,107]. These findings identify *NPM1*-mutated AML, and particularly *NPM1*/*FLT3* co-mutated disease, as a biologically attractive setting for CD123-directed immunotherapies, including CAR-T-cell approaches [105,107]. However, the expression of CD123 on non-leukemic cells, including endothelial cells, raises concerns regarding potential on-target/off-tumor toxicity. This has prompted the development of strategies aimed at increasing leukemic selectivity, including dual-targeting approaches combining CD123 with CD33 or other AML-associated antigens [108].

## 5. Targeting Core NPM1c-Dependent Oncogenic Pathways: The NPM1c–HOX/MEIS1 Transcriptional Program

Despite the genetic and biological heterogeneity of *NPM1*-mutated AML, a common feature of this disease is the maintenance of a distinctive *HOX*/*MEIS1*-high transcriptional program. In 1999, Lawrence et al. documented frequent co-expression of *HOXA9* and *MEIS1* in human myeloid leukemias, supporting the concept that their coordinated activation represents a recurrent leukemogenic program [109]. Subsequent studies established the functional cooperation between *HOXA* genes and *MEIS1* in promoting self-renewal and blocking myeloid differentiation [110,111,112]. *KMT2A*-rearranged and *NPM1*-mutated AML share a characteristic *HOX*/*MEIS1*-high gene-expression profile, although this transcriptional state is sustained through distinct upstream molecular mechanisms [10,113].

A major mechanistic advance came from Brunetti, Gundry et al., who demonstrated that the presence of mutant NPM1 in the cytoplasm (NPM1c) is required to maintain the leukemic state [10]. XPO1-inhibitor-mediated nuclear relocalization or targeted degradation of NPM1c resulted in rapid downregulation of *HOX*/*MEIS1* expression, followed by morphological and immunophenotypic differentiation and proliferative arrest in *NPM1*-mutated AML cell lines, patient-derived xenografts, and primary samples, irrespective of the co-mutational background [10]. Conversely, enforced *HOXA9*/*MEIS1* expression partially rescued the differentiation phenotype, identifying this transcriptional program as a critical downstream effector of NPM1c [10].

More recently, mutant NPM1 was shown to directly bind specific chromatin targets co-occupied by KMT2A, with targeted NPM1c degradation resulting in rapid loss of RNA polymerase II occupancy, activating histone modifications, and expression of leukemogenic target genes [114]. These findings provided a direct mechanistic link between mutant NPM1 and oncogenic transcription and further explained the dependency of *NPM1*-mutated AML on the menin–KMT2A complex.

Together, these observations identify the NPM1c–HOX/MEIS1 program as a central disease-maintenance dependency. Because direct pharmacological targeting of mutant NPM1 remains challenging, the most advanced therapeutic strategies exploit molecular mechanisms required to maintain this transcriptional state, particularly the menin–KMT2A interaction and XPO1-dependent nuclear export.

### 5.1. Menin Inhibition in NPM1-Mutated AML

Complementary preclinical studies provided pharmacological evidence for menin–KMT2A dependencies in *NPM1*-mutated AML. VTP-50469 reversed *NPM1c*-driven aberrant self-renewal, including in an *Npm1c*/*Dnmt3a*-mutated model, and showed activity against established *NPM1*-mutated AML [115]. Subsequently, MI-3454 induced differentiation and profound antileukemic responses, including complete remissions, in *NPM1*-mutated AML models and patient-derived xenografts [116].

These findings rapidly translated into clinical development. In the phase 1/2 AUGMENT-101 study, revumenib achieved a CR+CRh rate of 23.4% and an ORR of 46.9% in heavily pretreated patients with relapsed/refractory (R/R) *NPM1*-mutated AML [117]. Similarly, in the registration-enabling phase II KOMET-001 trial, ziftomenib achieved a CR+CRh rate of 22% and an ORR of 33%, with activity maintained across clinically relevant subgroups, including patients previously exposed to venetoclax [118].

Although these results establish menin inhibition as an effective targeted strategy in *NPM1*-mutated AML, responses to single-agent therapy are not uniformly durable. Acquired *MEN1* mutations affecting residues within the menin inhibitor-binding interface have emerged as an important on-target resistance mechanism, reducing inhibitor binding while preserving menin’s interaction with KMT2A [119].

These observations have prompted the development of combination strategies aimed at increasing response depth and durability. Early-phase studies combining menin inhibitors with azacitidine/venetoclax or intensive chemotherapy have shown encouraging activity [120,121], and this strategy is now being evaluated in randomized frontline trials in newly diagnosed *NPM1*-mutated AML (NCT05735184).

MEN1-independent mechanisms of resistance have also been described [122,123]. Preclinical studies have shown that disruption of chromatin-repressive complexes, including loss of PRC1.1 components (e.g., BCOR or PCGF1), can restore MYC and other noncanonical menin-regulated genes despite continued menin inhibition [122]. Resistant AML models without *MEN1* mutations may also undergo epigenetic reprogramming and develop alternative dependencies on BRD4 and the SWI/SNF ATPases SMARCA4/BRG1 and SMARCA2/BRM [123]. Thus, chromatin plasticity may provide alternative routes of escape from menin dependency, although its clinical relevance, particularly in *NPM1*-mutated AML, remains unclear.

Menin inhibitors are generally manageable from a safety perspective, with differentiation syndrome representing the most characteristic class-specific toxicity and requiring prompt recognition and treatment [117,118]. Regulatory translation has also been rapid: in 2025, the FDA approved revumenib and ziftomenib for defined populations with R/R *NPM1*-mutated AML, whereas neither agent currently has a marketing authorization in the European Union (Figure 1).

### 5.2. Targeting Nuclear Export in NPM1-Mutated AML

This NPM1c-dependent transcriptional state can also be therapeutically disrupted through XPO1 inhibition, which forces NPM1c nuclear relocalization and suppresses *HOX*/*MEIS1* expression [10].

Selinexor, a first-generation selective XPO1 inhibitor, was the first compound of this class evaluated clinically in AML [124]. However, despite a strong biological rationale and promising preclinical activity, clinical responses in *NPM1*-mutated AML were limited [124,125,126,127]. A major limitation is its toxicity profile, particularly nausea, anorexia, and fatigue, which precludes frequent dosing. Selinexor is therefore generally administered intermittently, once or twice weekly [124,125,126,127]. Given its relatively short plasma half-life of approximately 6 h, this schedule results in only transient XPO1 inhibition. During drug-free intervals, XPO1 activity recovers, allowing NPM1c to relocalize to the cytoplasm and the *HOX*/*MEIS1* transcriptional program to be re-established [11]. Preclinical studies showed that sustained XPO1 inhibition is instead required for durable *HOX*/*MEIS1* downregulation and terminal differentiation of *NPM1*-mutated AML cells [11].

Second-generation XPO1 inhibitors have been developed to overcome these limitations. Eltanexor, in particular, exhibits reduced central nervous system penetration, translating into a more favorable toxicity profile [128]. This enables more frequent dosing schedules, typically five days per week, and therefore more sustained XPO1 inhibition [11]. In *NPM1*-mutated AML models, prolonged eltanexor exposure resulted in stable *HOX*/*MEIS1* downregulation, terminal differentiation, reduced leukemic engraftment, and prolonged survival, providing the biological rationale for its clinical evaluation in this molecular subtype [11]. Eltanexor is currently being investigated in a dedicated phase II study in *NPM1*-mutated AML (ELENA-01; EU CT number 2024-514724-16-01).

Together, menin and XPO1 inhibition illustrate how shared NPM1c-dependent transcriptional dependencies can be therapeutically exploited across different co-mutational backgrounds.

## 6. Conclusions

Over the past two decades, *NPM1*-mutated AML has evolved from a molecularly recognizable subtype with relatively favorable clinical features into a disease-defining entity with a distinctive biology, a validated molecular marker for MRD monitoring, and an expanding spectrum of therapeutic vulnerabilities. Treatment selection is now increasingly shaped by patient fitness, co-mutational profile, and dynamic molecular response. Intensive chemotherapy remains a standard frontline approach for younger and fit patients, with the addition of gemtuzumab ozogamicin or FLT3 inhibitors in appropriate molecular settings, whereas venetoclax-based regimens and IDH-directed therapies have broadened treatment options for older or unfit patients. More recently, menin inhibition has provided the first clinically validated strategy directly exploiting a core transcriptional dependency of *NPM1*-mutated AML, while additional approaches such as XPO1 inhibition remain under clinical investigation (Figure 2).

Importantly, the work reviewed here also highlights the limitations of relying on baseline genomic classification alone. Co-occurring mutations contribute substantially to disease heterogeneity, but their prognostic impact is context-dependent and may be modified by targeted therapies and, critically, by post-treatment molecular response. Post-treatment *NPM1* MRD can therefore substantially refine the risk stratification provided by static baseline genetics. At the same time, even within ELN favorable-risk *NPM1*-mutated AML, a subset of patients remains at increased risk of treatment failure and relapse.

The next challenge is therefore to move beyond the simple cataloging of co-mutations toward the identification of integrated biological states that determine treatment response and relapse. Emerging transcriptomic, single-cell and multiomic studies indicate that *NPM1*-mutated AML comprises biologically distinct cellular and immune states that are not fully captured by conventional genomic risk models. Integration of baseline genetics with longitudinal MRD assessment and broader transcriptional, cellular, metabolic and microenvironmental information may ultimately provide a more accurate framework for risk stratification and therapeutic selection.

In this perspective, the *HOX*/*MEIS1*-high transcriptional state represents a paradigmatic example of how a shared biological dependency can transcend co-mutational heterogeneity and be therapeutically exploited. The future of precision medicine in *NPM1*-mutated AML will therefore likely rely on combining genotype, dynamic molecular response and disease-state biology to identify not only which patients are at risk, but also the mechanisms responsible for treatment failure and the therapeutic vulnerabilities that can be targeted to overcome it.

## Figures and Tables

**Table 1:** Summary of the major *NPM1* mutation types identified in AML [3,31].

Type of Mutation	Frequency	Nucleotide Sequence (Exon 12) *	Predicted C-Terminus
None (wild type)		GATCTCTGGCAGTGGAGGAAGTCTCTTTAA	–DLWQWRKSL
Mutation A	~75–80%	GATCTCTG**TCTG**GCAGTGGAGGAAGTCTCTTTAA	–DLCLAVEEVSLRK
Mutation B	~5–6%	GATCTCTG**CATG**GCAGTGGAGGAAGTCTCTTTAA	–DLCMAVEEVSLRK
Mutation C	~2%	GATCTCTG**CGTG**GCAGTGGAGGAAGTCTCTTTAA	–DLCVAVEEVSLRK
Mutation D	~5–10%	GATCTCTG**CCTG**GCAGTGGAGGAAGTCTCTTTAA	–DLCLAVEEVSLRK
Mutation E	~2%	GATCTCTGGCAGT**CTCTTGCCC**GGAGGAAG	–DLWQSLAQVSLRK
Mutation F	~2%	GATCTCTGGCAGT**CCCTGGAGA**GGAGGAA	–DLWQSLEKVSLRK
Mutation G	rare	GATCTCTG**CAGG**GCAGTGGAGGAAGTCTCTTTAA	–DLCRAVEEVSLRK
Mutation K	rare	GATCTCTG**CCGG**GCAGTGAGGAAGTCTCTTTAAGAA	–DLCRAVEEVSLRK
Mutation L	rare	GATCTCTG**CCGCGG**AGTGGAGGAAGTCTCTTTAAG	–DLCRGVEEVSLRK
Mutation M	rare	GATCTCTGGCAG**AGGA**TGGAGGAAGTCTTCTTTAAGAA	–DLWQRMEEVSLRK
Mutation N	rare	GATCTCTG**CCAG**GCAGTGGAGGAAGTCTCTTTAAGAA	–DLCQAVEEVSLRK
Mutation O	rare	GATCTCTG**TTTG**GCAGTGGAGGAAGTCTCTTTAAGAA	–DLCLAVEEVSLRK

* Bold formatting highlights the newly inserted nucleotides in the exon 12 sequence.

## Data Availability

No new data were created or analyzed in this study. Data sharing is not applicable to this article.

## Abbreviations

The following abbreviations are used in this manuscript:

AML	Acute Myeloid Leukemia
allo-HSCT	allogeneic Hematopoietic Stem Cell Transplantation
APL	Acute Promyelocytic Leukemia
Aza	Azacitidine
BM	Bone Marrow
BCOR	BCL6 Corepressor
BRD4	Bromodomain-Containing Protein 4
BRG1	Brahma-Related Gene 1
BRM	Brahma
CAR-T	Chimeric Antigen Receptor T-Cell
CR	Complete Remission
CRc	Composite Complete Remission
CRh	Complete Remission with partial hematologic recovery
dPCR	digital Polymerase Chain Reaction
EFS	Event-Free Survival
ELN	European LeukemiaNet
EOT	End Of Treatment
FLT3	FMS-Like Tyrosine Kinase 3
FLT3-ITD	FLT3 Internal Tandem Duplication
FLT3i	FLT3 inhibitor
GO	Gemtuzumab Ozogamicin
ICC	International Consensus Classification
IHC	Immunohistochemistry
KMT2A	Lysine Methyltransferase 2A
MFC	Multiparameter Flow Cytometry
MR	Myelodysplasia-related
MRD	Measurable Residual Disease
MYC	MYC Proto-Oncogene
NES	Nuclear Export Signal
NGS	Next-Generation Sequencing
NoLS	Nucleolar Localization Signal
NPM1	Nucleophosmin 1
ORR	Overall Response Rate
OS	Overall Survival
PB	Peripheral Blood
PCR	Polymerase Chain Reaction
PCGF1	Polycomb Group Ring Finger Protein 1
PRC1.1	Polycomb Repressive Complex 1.1
qPCR	quantitative Polymerase Chain Reaction
R/R	Relapsed/Refractory
SMARCA2/4	SWI/SNF-Related, Matrix-Associated, Actin-Dependent Regulator of Chromatin, Subfamily A, Member 2/4
SWI/SNF	SWItch/Sucrose Non-Fermentable chromatin-remodeling complex
UHS-NGS	Ultrahigh-Sensitivity Next-Generation Sequencing
Ven	Venetoclax
XPO1	Exportin 1
